# Synthesis and Properties of Tung Oil-Based Unsaturated Co-Ester Resins Bearing Steric Hindrance

**DOI:** 10.3390/polym11050826

**Published:** 2019-05-07

**Authors:** Chengguo Liu, Qiong Wu, Rongrong An, Qianqian Shang, Guodong Feng, Yun Hu, Puyou Jia, Yonghong Zhou, Wen Lei

**Affiliations:** 1 National Engineering Lab for Biomass Chemical Utilization; Key Lab of Chemical Engineering of Forest Products, National Forestry and Grassland Administration; Key Lab of Biomass Energy and Material, Jiangsu Province; Co-Innovation Center of Efficient Processing and Utilization of Forest Resources, Jiangsu Province; Institute of Chemical Industry of Forest Products, Chinese Academy of Forestry, Nanjing 210042, China; wqzjj9394@163.com (Q.W.); Shang_qianqian@126.com (Q.S.); fengguodong21@163.com (G.F.); 15150509893@139.com (Y.H.); jiapuyou@icifp.cn (P.J.); 2 College of Science, Nanjing Forestry University, Nanjing 210037, China; 3 College of Geographic and Biologic Information, Nanjing University of Posts and Telecommunications, Nanjing 210023, China; anrongrong@njupt.edu.cn

**Keywords:** tung oil, unsaturated polyester resins, thermosetting polymers, structure–property relationship, structural plastics

## Abstract

New tung oil (TO)-based, unsaturated, co-ester (Co-UE) macromonomers bearing steric hindrance were synthesized by modifying a TO-based maleate (TOPERMA) monomer with an anhydride structure with hydroxyethyl methacrylate (HEMA) and methallyl alcohol (MAA), respectively. The obtained Co-UE monomers (TOPERMA-HEMA and TOPERMA-MAA) were then characterized by ^1^H NMR and gel permeation chromatography (GPC). For comparison, hydroxyethyl acrylate (HEA)-modified TOPERMA (TOPERMA-HEA) was also synthesized and characterized. Subsequently, the obtained Co-UEs were thermally cured with styrene, and the ultimate properties of the resulting materials were studied. It was found that by introducing the structure of steric hindrance into the TO-based Co-UE monomer, the tensile strength and Young’s modulus of the resulting materials were improved. Furthermore, by reducing the length of the flexible chain in the Co-UE monomer, the tensile strength, Young’s modulus, and glass transition temperature (*T*_g_) of the resultant materials were also improved. The TOPERMA-MAA resin gave the best performance in these TO-based Co-UE resins, which showed a tensile strength of 32.2 MPa, Young’s modulus of 2.38 GPa, and *T*_g_ of 130.3 °C. The developed ecofriendly materials show promise in structural plastic applications.

## 1. Introduction

Unsaturated polyester resin (UPR) is one of the most frequently used thermosetting polymers because of its low price, ease of handling, and good balance of mechanical, thermal, electrical, and chemical properties. As the main component of fiber-reinforced plastics (FRPs) and some other composites, UPR has been widely utilized in the areas of transportation, marine applications, pipes and tanks, wind energy, construction, electronics, etc [1,2,3,4]. However, due to the uncertainties about petroleum’s supply and price in the future as well as environmental problems, substitutes of polymers from natural resources such as carbohydrates, lignin, and natural oils have been intensively explored [5,6,7,8,9,10,11,12]. Among all the bioresources, plant oils are the most common one employed for the development of UPRs thanks to their abundance and low cost, triglyceride structure suitable for further chemical manipulation, and biodegradability [13,14]. The development of UPRs with plant oils may not only provide a means of sustainable development for the present UPR industry, but also can improve the value of plant oil resources.

Generally, two methods have been reported for chemically converting plant oils to UPRs. The first method is converting plant oils into feedstocks such as diacids and diols, which can generate linear unsaturated polyesters via direct polycondensation [15,16]. In fact, this method is not commonly used, possibly because plant oils are not easily converted into high-purity feedstocks, and the content of the resulting materials is low if only one kind of oil-based feedstock was employed. As a result, the second method, converting plant oils into unsaturated ester (UE) macromonomers or oligomers containing active C=C groups, became popular. By introducing maleic or acrylic moiety, a variety of plant oil-based UE monomers or oligomers were developed [17,18,19,20,21,22,23,24,25,26]. However, most of the obtained UEs possessed a low C=C functionality, thus leading to low cross-ink density and inferior performance in stiffness and heat resistance in the resulting materials. For instance, acrylated epoxidized soybean oil (AESO), synthesized through the ring-opening reaction of epoxidized soybean oil with acrylic acid, usually possessed a C=C functionality of 2–4. Consequently, the resultant AESO resin with 40 wt % of styrene only demonstrated a tensile strength of 21 MPa, Young’s modulus of 1.6 GPa, and glass transition temperature (*T*_g_) of 65 °C [17]. In order to improve the C=C functionality of AESO, maleic anhydride was used to further react with the generated hydroxyl groups on AESO [22]. The obtained MA-modified AESO (MAESO) could reach a C=C functionality up to 5.9, and the resulting materials with 38 wt % of styrene showed a tensile strength of 41−44 MPa, Young’s modulus of 2.2–2.5 GPa, and *T*_g_ of 115–130 °C. As the new UE monomer comprised two types of active C=C moieties, it can be named as an unsaturated co-ester (Co-UE). However, up until now, only a few routes were developed to acquire such UE monomers or oligomers.

In our previous work, in an effort to improve the C=C functionality of a tung oil (TO)-based maleate (TOPERMA) monomer bearing an anhydride structure, hydroxyethyl acrylate (HEA) was used to further modify the TOPERMA, and a new TO-based Co-UE macromonomer was obtained [27]. It should be noted that this was the first instance where to a Co-UE monomer was achieved by introducing maleic groups firstly and acrylic groups subsequently onto plant oils. At last, a novel tung oil-based Co-UE monomer (TOPERMA-HEA) was obtained, which had a tensile strength of 36.3 MPa, Young’s modulus of 1.70 GPa, and *T*_g_ of 127 °C after curing with 40 wt % of styrene. Although the properties of this new, biobased, Co-UE resin were good among all the reported oil-based UE resins, some key properties, like stiffness, were still far inferior to those of petroleum-based UPRs.

**Scheme 1:**
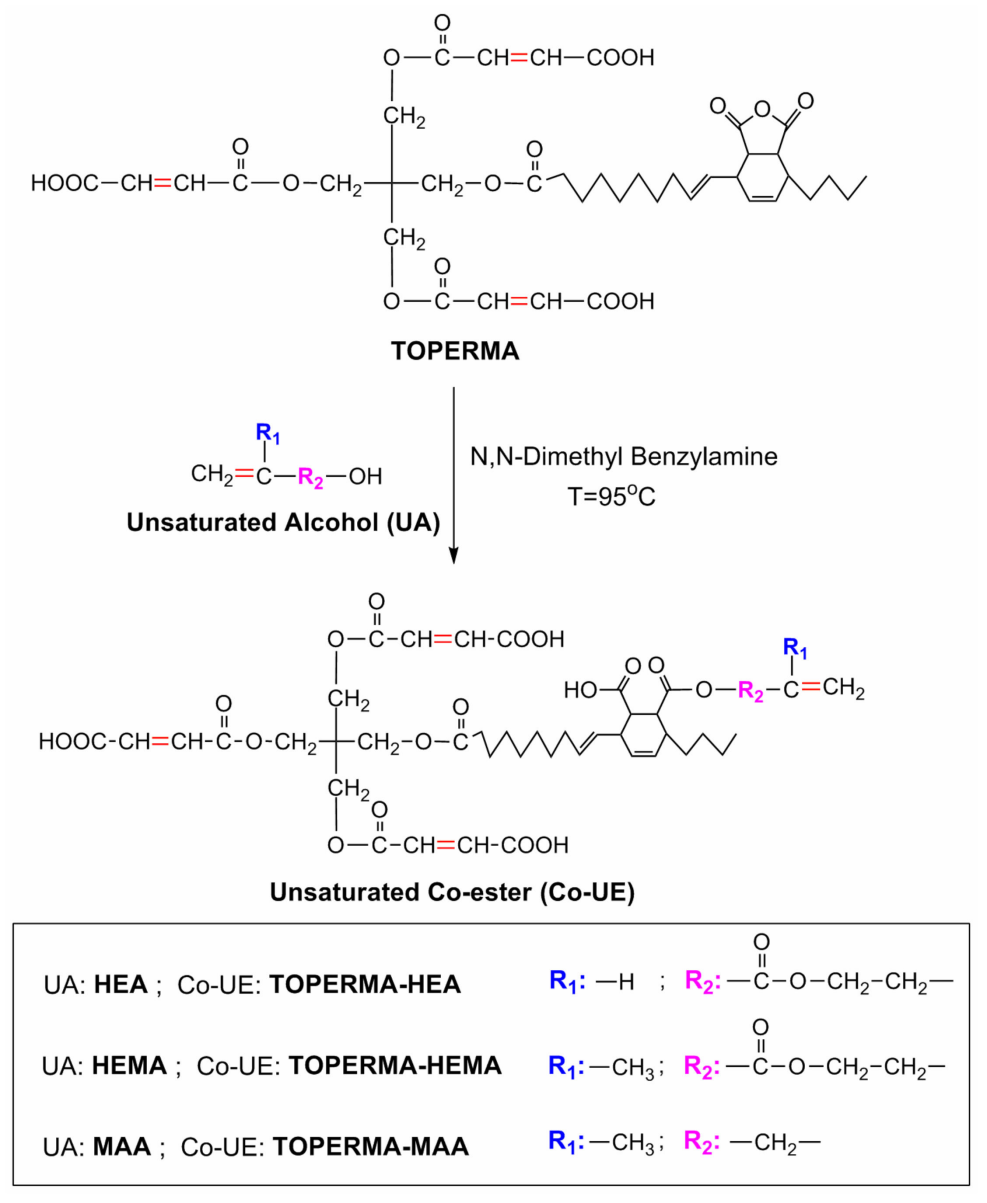
Synthesis of tung oil-based, unsaturated co-esters (Co-UEs) by modifying tung oil (TO)-based maleate (TOPERMA) with different unsaturated alcohols.

In order to achieve better performance of such biobased Co-UE resins, two novel, TO-based, Co-UE macromonomers were synthesized by modifying TOPERMA with hydroxyethyl methacrylate (HEMA) or methallyl alcohol (MAA), as shown in Scheme 1. For comparison, HEA-modified TOPERMA (TOPERMA-HEA) and TOPERMA products were also synthesized. Another important goal for this study is to establish the structure–property relationship for the new, biobased, Co-UE resins. As indicated in Scheme 1, by modifying the types of unsaturated alcohol, two structural factors—steric hindrance (corresponding to the change of R_1_) and length of flexible chain (corresponding to the change of R_2_)—could be modulated, and their effects on the ultimate properties of the resultant biobased materials were carefully investigated.

## 2. Experimental

### 2.1. Materials

Tung oil (TO) was obtained from Jiangsu Donghu Bioenergy Plant Plantation Co., Ltd. (Yancheng, China), which possessed a yellow color and specific gravity of 0.935−0.940 at 25 °C. The pentaerythritol (PER, ≥98%) and maleic anhydride (MA, ≥99.5%) solid were obtained from Nanjing Chemical Reagent Co., Ltd. (Nanjing, China). The HEA liquid (≥97%) was supplied by Macklin Chemical Reagent Co., Ltd. (Shanghai, China). The HEMA liquid (≥97%) was supplied by Adamas Reagent Co., Ltd. (Shanghai, China). The MAA liquid (≥97%) was supplied by Shanghai Energy Chemical Co., Ltd. (Shanghai, China). Calcium hydroxide (Ca(OH)_2_, ≥95%) and *N*,*N*-dimethyl benzyl amine (≥98%) were purchased from Tianjin Chemical Reagent Institute Co., Ltd. (Tianjin, China). Hydroquinone (≥99%) and styrene (≥99%) were purchased from Chengdu Kelong Chemical Reagent Co., Ltd. (Chengdu, China). The initiator *tert*-butyl peroxy benzoate (*t*-BPB, ≥ 98%) was obtained from Shanghai Aladdin Chemistry Co., Ltd. (Shanghai, China). The styrene, HEA, HEMA, MAA, and *N*,*N*-dimethyl benzyl amine were all dried by molecular sieves for at least 1 week before use.

### 2.2. Synthesis of TOPERMA

The TO-based maleate was synthesized according to our previous work [28]. Typically, about 348.8 g of TO, 108.8 g of PER, and 4.6 g of Ca(OH)_2_ were placed together in a 1 L, four-neck, round-bottom flask equipped with a mechanical stirrer, thermometer, nitrogen (N_2_) gas inlet, and refluxing condenser capped with an anhydrous calcium chloride drier. The reaction mixture was heated to about 230 °C by a heating mantle under N_2_ atmosphere and agitated at this temperature for 2 h. After 2 h, the mixture was cooled using ice water, and the tung oil pentaerythritol alcoholyis (TOPER) product was obtained. For the maleinization reaction, 231.1 g of the obtained TOPER product, 176.5 g of MA, and 0.82 g of hydroquinone were added to the same reaction device as that in the alcoholysis process. The mixture was heated to 70 °C by an oil bath and agitated at this temperature until the MA completely melted and mixed with TOPER. After that 4.08 g of *N*,*N*-dimethyl benzylamine was added and the reaction mixture was heated to 95 °C under N_2_ atmosphere. The mixture was agitated at this temperature for 5h. Finally, crude, maleinated product (TOPERMA) was obtained. The product was divided into two parts: a small part was purified, while the left large part was employed to further synthesize TO-based Co-UEs. In the purifying process, the product was put into a separating funnel and washed three times with hot saturated sodium chloride (NaCl)/water solution (prepared by blending sodium chloride with boiled water). Subsequently, dichloromethane was added to dissolve the product, and the separated organic layer was dried by anhydrous magnesium sulfate overnight. Finally, the solution was filtered and the solvent was removed by a rotary evaporator. The purified TOPERMA product (with a yield of 66.9%) was a light brown and transparent liquid resin when cooled naturally to room temperature.

### 2.3. Synthesis of TO-Based Co-UEs

Firstly, around 100 g of the unpurified TOPERMA product was added into a 250 mL, four-neck, round-bottom flask. It should be stated that the unpurified product (~350 g) mentioned above was added into three 250 mL flasks at a time to avoid repeatedly heating the product. Then, about 8.71 g (0.075 mol) of HEA, 9.76 g of HEMA (0.075 mol), or 5.41 g (0.075 mol) of MAA was added to the flask, and the device was also equipped with a mechanical stirrer, thermometer, N_2_ gas inlet, and refluxing condenser with an anhydrous calcium chloride drier. The reaction mixture was heated to 95 °C under N_2_ atmosphere and agitated at this temperature for 4 h. The obtained products were purified with the same procedure as that of the TOPERMA product. Finally, three TO-based, Co-UE products were obtained, which were all light brown and transparent liquid resins at room temperature. According to the unsaturated alcohols used, the products were labeled as TOPERMA-HEA, TOPERMA-HEMA, and TOPERMA-MAA. The yields of TOPERMA-HEA, TOPERMA-HEMA, and TOPERMA-MAA were 73.0%, 79.3%, and 78.5%, respectively.

### 2.4. Curing of the TO-Based Resins

All of the obtained TO-based resins were cured following the same procedure. First, the TO-based resin samples were prepared by blending the TO-based macromonomer, styrene (40% of total weight of the macromonomer and styrene), and hydroquinone (0.2% of the total weight of the macromonomer and styrene) for 30 min at room temperature. The obtained samples were then mixed with *t*-BPB initiator (3% of total weight of the macromonomer and styrene) for 30 min, degassed at 40 °C for 10 min, and poured into homemade polytetrafluoroethylene molds (the sketch and description were shown in Figures S1 and S2). Finally, the samples in the molds were thermally cured at 80 °C for 2 h, 120 °C for 2 h, and postcured at 160 °C for 1 h.

### 2.5. Characterization

#### 2.5.1. Gel Permeation Chromatography (GPC)

The GPC tests of samples were conducted on a high-pressure liquid chromatography (HPLC) system (Waters Corporation, Milford, MA, USA) equipped with a 1515 isocratic HPLC pump, 717 plus automated injector, and column heater. The separating columns were a pair of Styragel HR1 and HR2 (300 mm × 7.8 mm), and the temperature of the columns was 35 °C. The ranges of molar masses for the HR1 and HR2 columns were 100−5000 and 500−20,000, respectively. A Waters 2414 Refractive Index (RI) detector was employed to detect RI signals. Chromatographic-grade tetrahydrofuran (THF) was employed as an eluent with a flow rate of 1.0 mL/min. The samples were dissolved by THF in disposable transparent glass vials with a concentration of 15−25 mg/mL. The relative molar masses of samples were determined with a standard curve calibrated by a series of narrowly distributed polystyrene standards with a known molar mass of 580−19,600.

#### 2.5.2. Nuclear Magnetic Resonance (NMR)

The ^1^H NMR spectra of samples were recorded on a DRX-300 Advance NMR spectrometer (Bruker Corporation, Billerica, MA, USA) using CDCl_3_ as the solvent.

#### 2.5.3. Dynamic Mechanical Analysis (DMA)

The DMA tests of the cured samples were conducted on a Q800 solids analyzer (TA Corporation, New Castle, DE, USA) with a three-point bending geometry and an oscillating frequency of 1 Hz. All the samples were polished carefully to avoid surface defects before the test. The samples with a size of 40 × 10 × 4 mm^3^ were examined at a heating rate of 3 °C∙min^−1^.

#### 2.5.4. Thermogravimetric Analysis (TGA)

The TGA tests of the cured samples were carried out on an STA 409PC thermogravimetry instrument (Netzsch Corporation, Selb, Germany) with a heating rate of 15 °C∙min^−1^. Ground samples with a mass of about 10 mg were heated in a range of 35–600 °C under N_2_ gas at a flow rate of 100 mL/min.

#### 2.5.5. Mechanical Properties

The tensile tests of the cured samples were evaluated on a SANS7 CMT-4304 universal tester from Shenzhen Xinsansi Jiliang Instrument Corporation (Shenzhen, China) at a crosshead speed of 5.0 mm/min. Dumbbell specimens with a size of 9.53 × 3.18 × 3.2 mm^3^ at the narrow middle part and an overall length of 63.5 mm were conducted for the tensile tests. All the specimens were polished to avoid surface defects before the test. For each sample, at least five specimens were tested to determine the average values.

#### 2.5.6. Water Absorption

The cured samples were dried in an oven at 50 °C for 24 h and weighed. Subsequently, the dried samples were immersed into distilled water for 48 h at room temperature. At last, the immersed samples were dried with filter papers and weighed again. For each resin, two samples were tested to determine the average values. The water absorption was calculated based on the following equation:

$$S=\frac{W1-W0}{W1}\times 100\%$$

where *W*_0_ and *W*_1_ are the weights of the sample before and after being immersed into water, respectively.

## 3. Results and Discussion

### 3.1. Characterization of TO-Based Co-UEs

**Figure 1:**
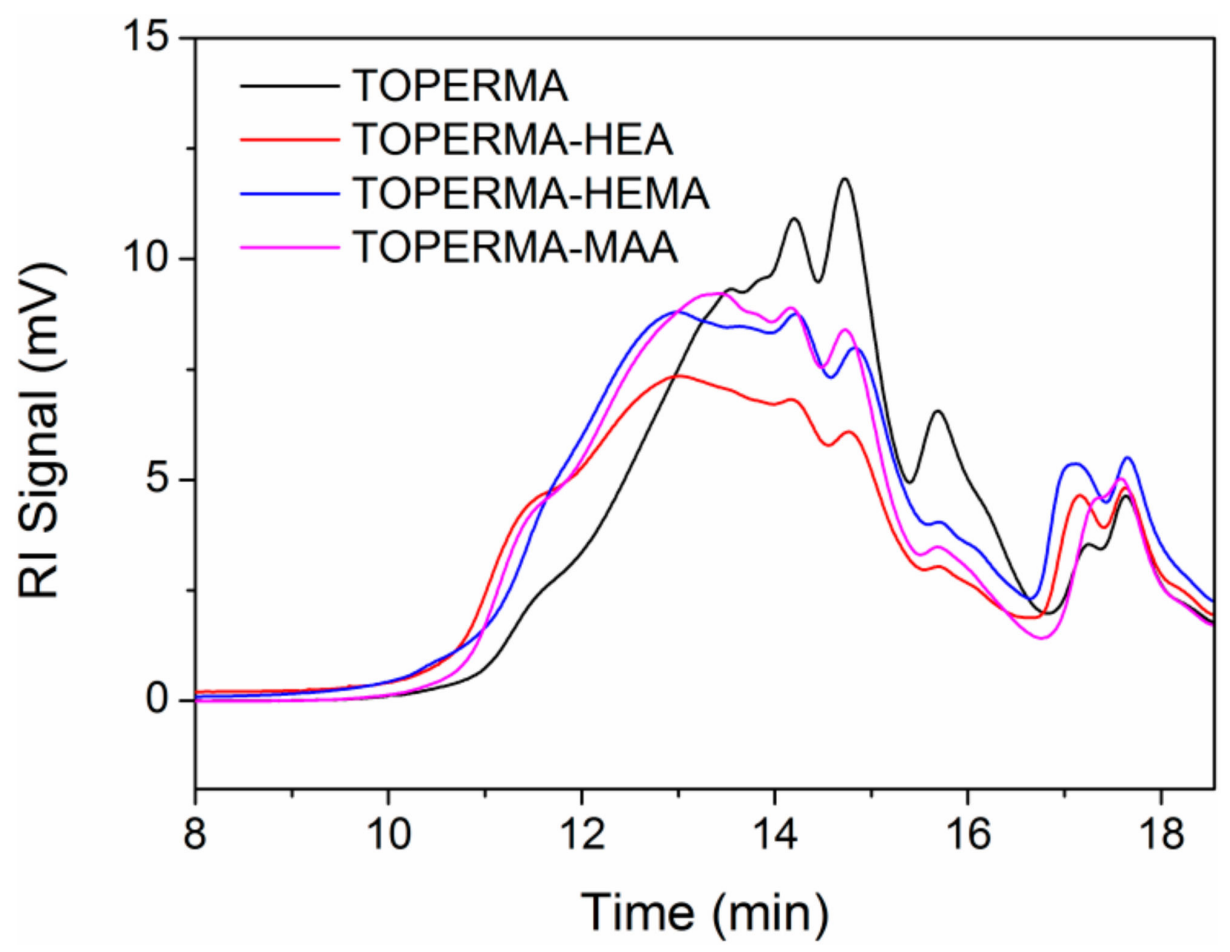
Gel permeation chromatography (GPC) chromatographs of TO-based maleate and Co-UEs.

Figure 1 shows the GPC chromatographs of TO-based maleate and Co-UEs. Multi-peaks were observed in the chromatograph of TOPERMA, as the TOPERMA product was composed of mixed TO-based maleates [28]. This phenomenon was also seen in the chromatographs of TO-based Co-UEs. Furthermore, all the chromatographs of TO-based Co-UEs moved to a shorter retention time compared with TOPERMA, indicating the growth of the molar masses for all the Co-UE monomers [29]. Using the calibration curve of polystyrene standards, the molar masses of the TOPERMA and Co-UE monomers were calculated and listed in Table 1. All the TO-based Co-UEs demonstrated higher weight-average and number-average molar masses (*M*_w_ and *M*_n_) than TOPERMA, indicating the unsaturated alcohols, including HEA, HEMA, and MAA, were successfully grafted onto TOPERMA.

**Figure 2:**
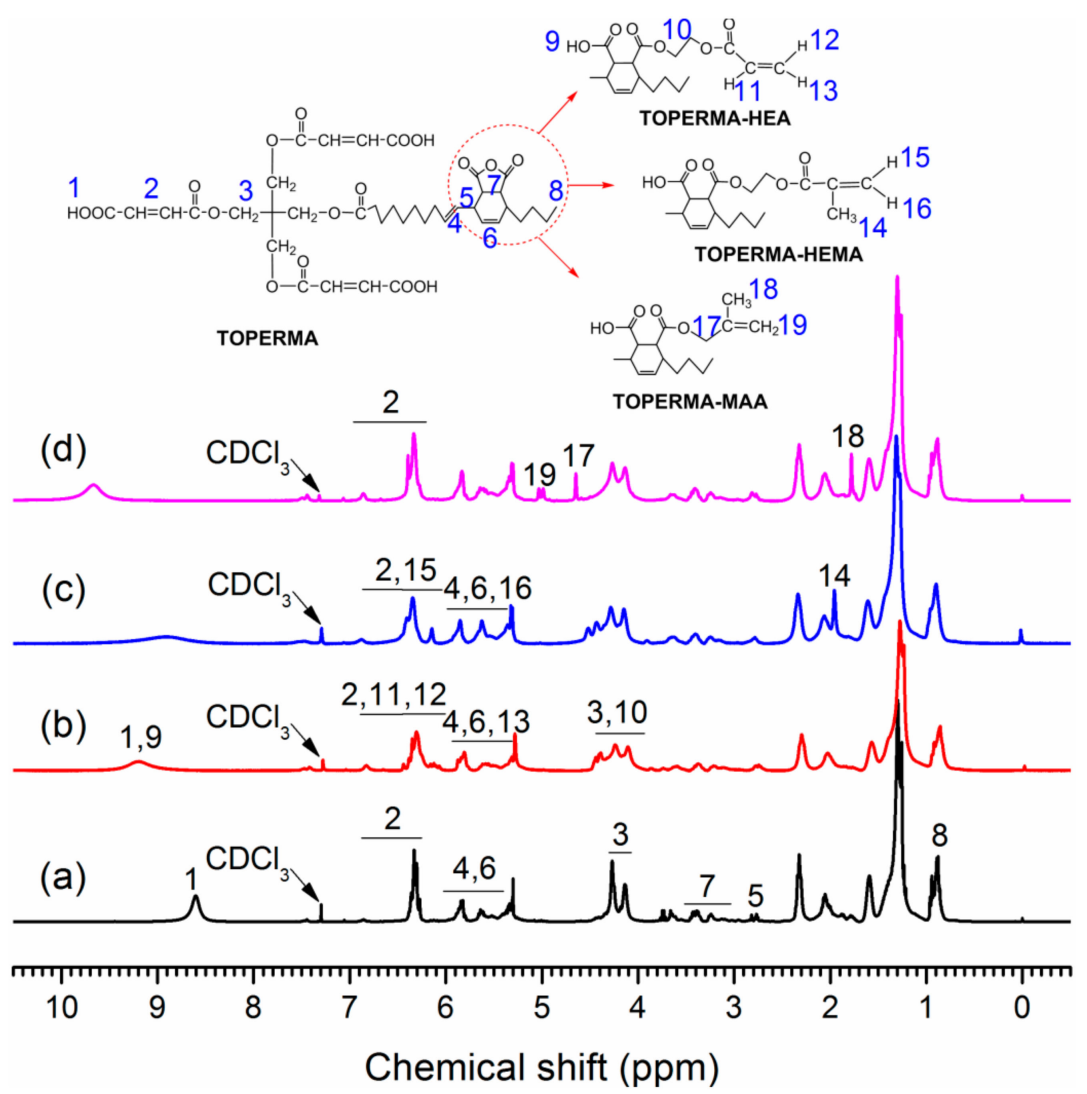
^1^H NMR spectra of (**a**) TOPERMA, (**b**) TOPERMA-HEA, (**c**) TOPERMA-HEMA, and (**d**) TOPERMA-MAA.

Figure 2 indicates the ^1^H NMR spectra of TO-based maleate and Co-UEs. In the spectrum of TOPERMA, the wide peak at 8.6 ppm corresponded to the protons on the carboxyl groups. The peaks at 6.2–6.9 ppm were corresponding to the maleate and fumarate vinyl protons. The multi-peaks at 5.3–5.9 ppm represented the residue protons of TO-conjugated trienes and the protons of the new yielding C=C due to D–A addition. The peaks at 4.0–4.4 ppm were assigned to the methylene protons of the polyol backbones connecting to the ester structure. The low peaks at 3.1–3.5 ppm represented the protons in the structure where MA connected to TO-conjugated triene. The peak at around 0.9 ppm corresponded to the terminal methyl protons of TO fatty acids, which is always taken as a reference to calculate proton amounts because its intensity should not be altered throughout all the reactions. In the spectrum of TOPERMA-HEA, the peak related to the protons on carboxyl groups shifted to 9.2 ppm and widened due to newly generated carboxyl groups. New and low peaks occurred at around 6.1 ppm, which was assigned to two vinyl protons on acrylate [27]. New peaks also appeared at about 5.8 ppm and 4.3–4.5 ppm, corresponding to the left vinyl protons on acrylate and the methylene protons from HEA, respectively. In the spectrum of TOPERMA-HEMA, the peak about the protons on carboxyl groups shifted to 8.9 ppm and also widened. New sharp peaks occurred at around 6.1 ppm, which was assigned to one of the two vinyl protons on methacrylate. New peaks appeared at around 2.0 ppm, which corresponded to the methyl protons on methacrylate. In the spectrum of TOPERMA-MAA, the peak corresponding to the protons on the carboxyl groups shifted to 9.7 ppm. New peaks occurred at around 5.0, 4.6, and 1.8 ppm, which were assigned to the two vinyl protons, methylene protons, and methyl protons on the methallyl groups, respectively. In general, all of these changes indicated that TOPERMA was successfully modified by HEA, HEMA, and MAA, respectively.

Using the peak at around 0.9 ppm as a reference, the proton amounts of the TO-based products can be determined. Combined with the information on the vinyl protons mentioned above, the introduced C=C functionality per fatty acid (*N*_C=C_) for each product could be determined by the following equations:

(1) $$N_{C=C}(\mathrm{TOPERMA})=\frac{1}{2}(\frac{A_{6.2-6.9ppm}}{A_{0.9ppm}/3})$$

(2) $$N_{C=C}(\mathrm{TOPERMA-HEA})=\frac{1}{2}(\frac{A_{6.0-6.9ppm}}{A_{0.9ppm}/3})$$

(3) $$N_{C=C}(\mathrm{TOPERMA-HEMA})=\frac{1}{2}(\frac{A_{6.2-6.9ppm}}{A_{0.9ppm}/3})+\frac{A_{6.1ppm}}{A_{0.9ppm}/3}$$

(4) $$N_{C=C}(\mathrm{TOPERMA-MAA})=\frac{1}{2}(\frac{A_{6.2-6.9ppm}}{A_{0.9ppm}/3})+\frac{1}{2}(\frac{A_{5.0ppm}}{A_{0.9ppm}/3})$$

The results are summarized in Table 1. Firstly, all the *N*_C=C_ values of the TO-based Co-UEs were larger than that of TOPERMA, suggesting the successful graft of unsaturated alcohols onto TOPERMA too. The grafted amounts of HEA, HEMA, and MAA were 0.36, 0.30, and 0.35, respectively. Second, the *N*_C=C_ values for the three TO-based Co-UEs were almost equal to each other, meaning that they are suited to be employed in the study of structure–property relationship for the new Co-UE resins.

### 3.2. Properties of TO-Based Co-UE Resins

#### 3.2.1. Dynamic Mechanical Analysis

**Figure 3:**
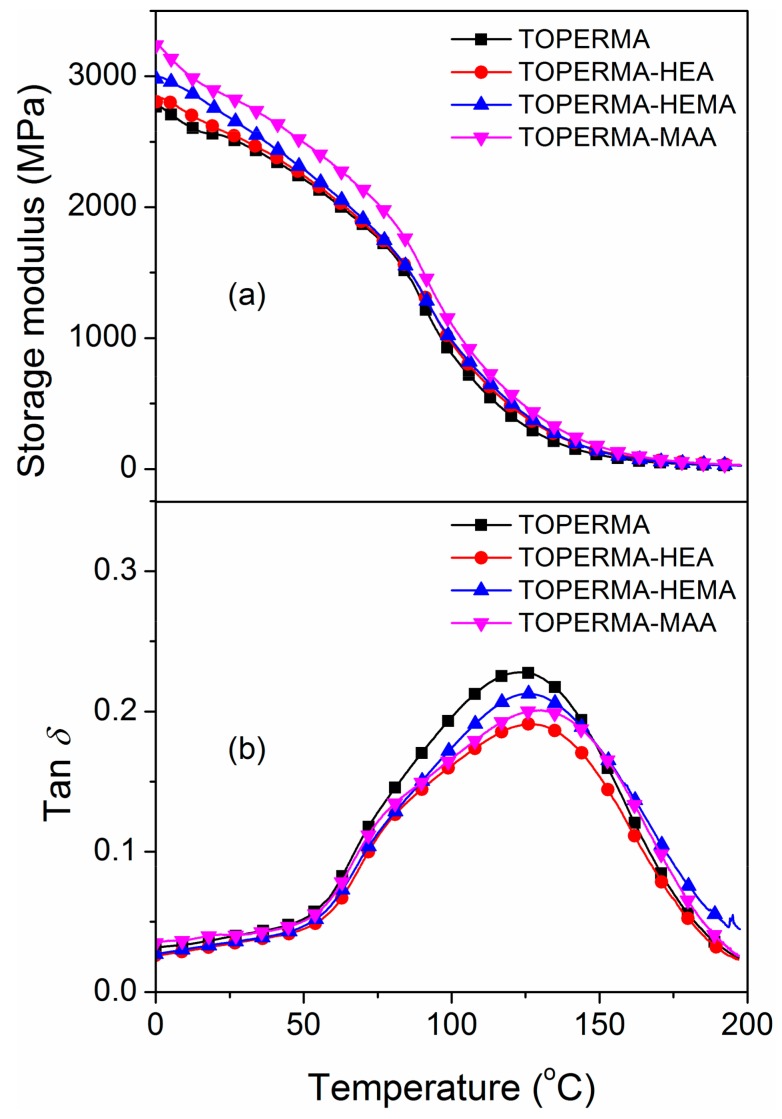
(**a**) Storage modulus and (**b**) loss factor of the cured, TO-based resins.

Figure 3 shows DMA curves of the cured TO-based maleate and Co-UE resins containing 40 wt% of styrene. The related data, including storage modulus at 25 °C (*E*′_25_) and *T*_g_ (determined from peak temperatures of tan δ curves), were summarized in Table 2. Compared with the TOPERMA resin, all the Co-UE resins demonstrated higher values of E′_25_ and *T*_g_, indicating that the incorporation of an unsaturated alcohol structure was beneficial to the improvement of the resin’s thermo-mechanical properties. Compared with the TOPERMA-HEA resin, *E*′_25_ value of the TOPERMA-HEMA resin increased by 0.04 GPa, while the *T*_g_ value decreased by 0.8 °C, suggesting that only the stiffness of the resin was improved by the incorporation of steric hindrance. Moreover, compared with the TOPERMA-HEMA resin, the *E*′_25_ and *T*_g_ values of the TOPERMA-MAA resin grew by 0.22 GPa and 5.3 °C, indicating that both the stiffness and heat resistance could be improved by the reduction of the length of the flexible chain.

The thermo-mechanical properties of thermosets usually have a close relationship with crosslink density (*ν_e_*). According to the kinetic theory of rubber elasticity, the experimental *ν_e_* of thermosets can be determined from the rubbery modulus by the following equation [22,27,30]

(5) $$E\prime =3\nu_{e}RT$$

where *E*′ represents the storage modulus of crosslinked copolymers in the rubbery plateau region, *R* is the gas constant, and *T* is the absolute temperature. In this work, the rubber modulus at *T*_g_ + 50 °C was selected for the calculation of *ν_e_*. It was observed that all of the *ν_e_* values of the obtained Co-UE resins were apparently larger than that of the TOPERMA resin. The reason for this lies in how all the Co-UEs had higher C=C functionality than TOPERMAA. In addition, in comparison with the TOPERMA-HEA resin, the drop in *T*_g_ for the TOPERMA-HEMA resin was probably attributed to the decrease of *ν_e_*.

#### 3.2.2. Thermogravimetric Analysis

**Figure 4:**
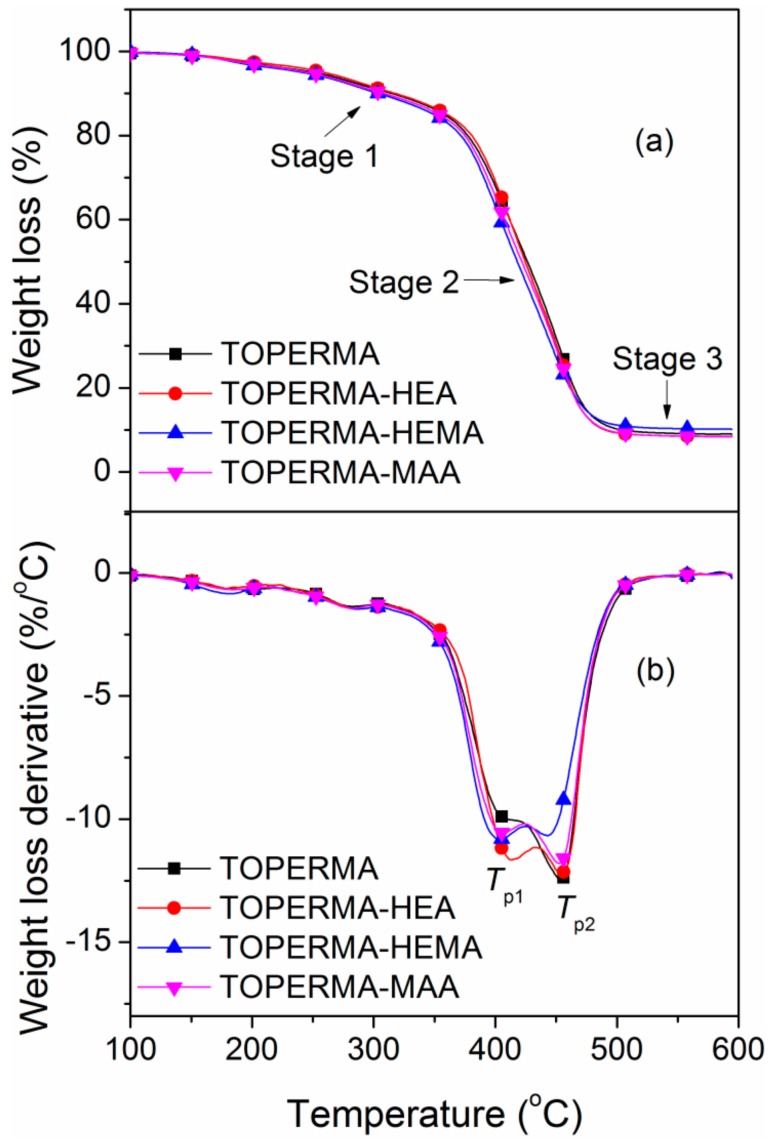
(**a**) TGA curves and (**b**) their derivatives of the cured TO-based resins.

Figure 4 depicts TGA thermograms and their derivative curves of the cured, TO-based maleate and Co-UE resins. All of the materials were thermally stable below 150 °C and exhibited a three-stage decomposition course [31]. The first stage was within 150−350 °C and can be ascribed to the evaporation and decomposition of soluble components (e.g., unreacted feedstocks) in the bulk materials. The second stage at about 350−500 °C was the fastest, which may result from the degradation and char formation of the crosslinked structures. The final stage (>500 °C) corresponded to the gradual degradation of char residue. The data of the obtained materials, including 5% weight loss temperature (*T*_5_), peak temperature at the curves of weight loss rate (*T*_p_), and char yield (*w*_char_), are listed in Table 2. Compared with the TOPERMA resin, the *T*_5_ and *T*_p_ values decreased slightly for almost all of the Co-UE resins, which may be ascribed to the flexible components from the unsaturated alcohols. However, the *T*_p_ values of the TOPERMA-HEA resin is an exception, which may be attributed to the large growth of *ν_e_*. Compared with the TOPERMA-HEA resin, all of the *T*_5_ and *T*_p_ values of the TOPERMA-HEMA resin decreased, which could be caused by the drop of *ν_e_*. Moreover, compared with the TOPERMA-HEMA resin, all of the *T*_5_ and *T*_p_ values of the TOPERMA-MAA resin increased, which is possibly ascribed to the reduction of the length of the flexible chain.

#### 3.2.3. Mechanical Properties

**Figure 5:**
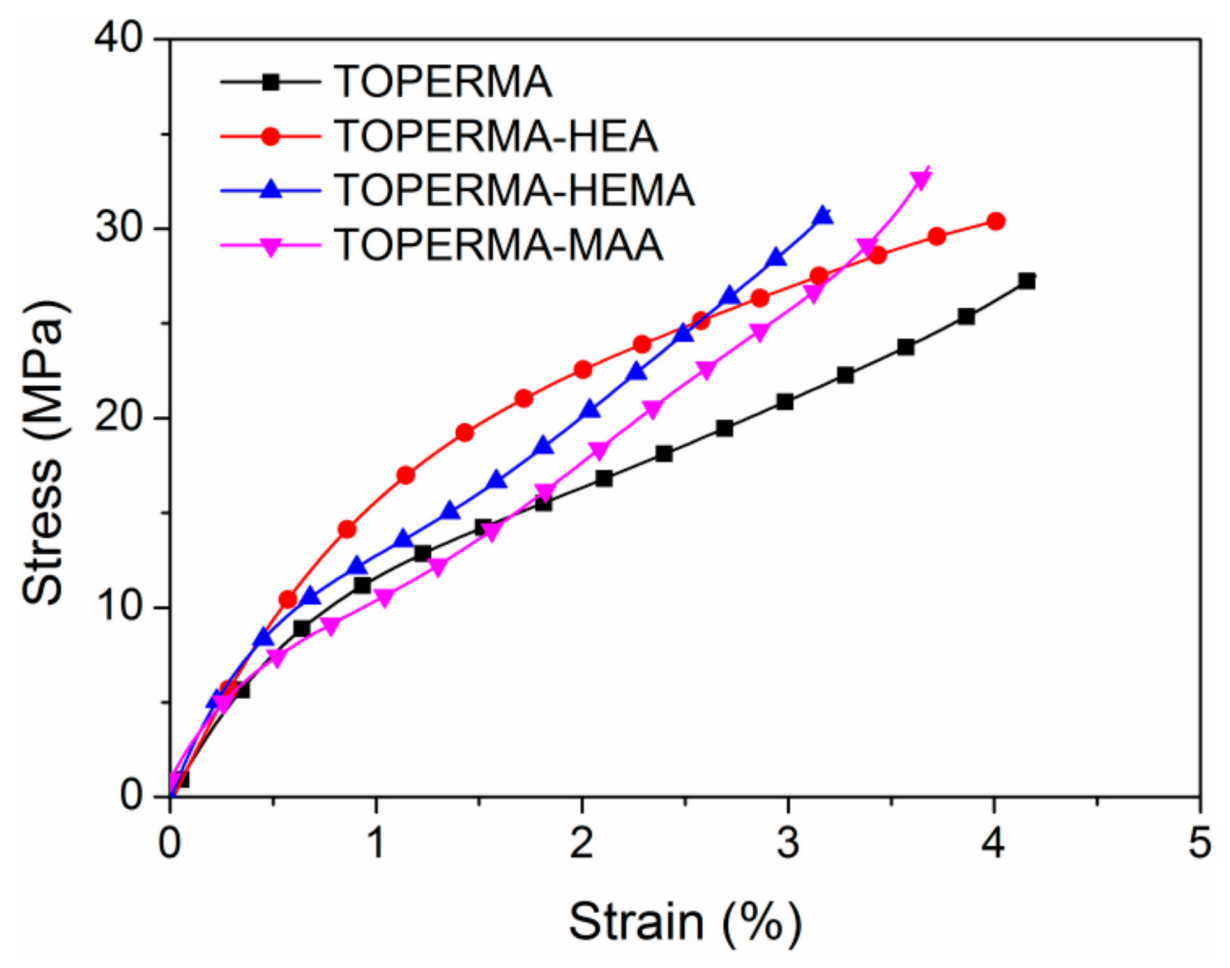
Typical tensile curves of the cured TO-based resins.

Figure 5 demonstrates the typical tensile stress–strain curves of the cured TO-based maleate and Co-UE resins. The corresponding data, including tensile strength (*σ*), Young’s modulus (*E*), and tensile breaking strain (*ε*), are summarized in Table 3. All of the TO-based materials broke without reaching a yielding point, indicating that they were rigid materials. Compared with the TOPERMA resin, the *σ* and *E* values for all the Co-UE resins increased, indicating that the stiffness of the new Co-UE resins was improved by the incorporation of unsaturated alcohols. Compared with the TOPERMA-HEA resin, the *σ* and *E* values for the TOPERMA-HEMA resin increased by 0.6 MPa and 0.10 GPa, suggesting that the stiffness of the Co-UE resins was improved by the incorporation of steric hindrance. Furthermore, compared with the TOPERMA-HEMA resin, the *σ* and *E* values of the TOPERMA-MAA resin increased by 1.3 MPa and 0.26 GPa, which means that the stiffness of the Co-UE resins was also improved by the reduction of the length of the flexible chain. The TOPERMA-MAA resin reached a maximum *σ* of 32.2 MPa and *E* of 2.38 GPa among the CO-UE resins, which is probably because it contained both the structures of steric hindrance and shortest length of the flexible chain.

#### 3.2.4. Water Absorption

**Figure 6:**
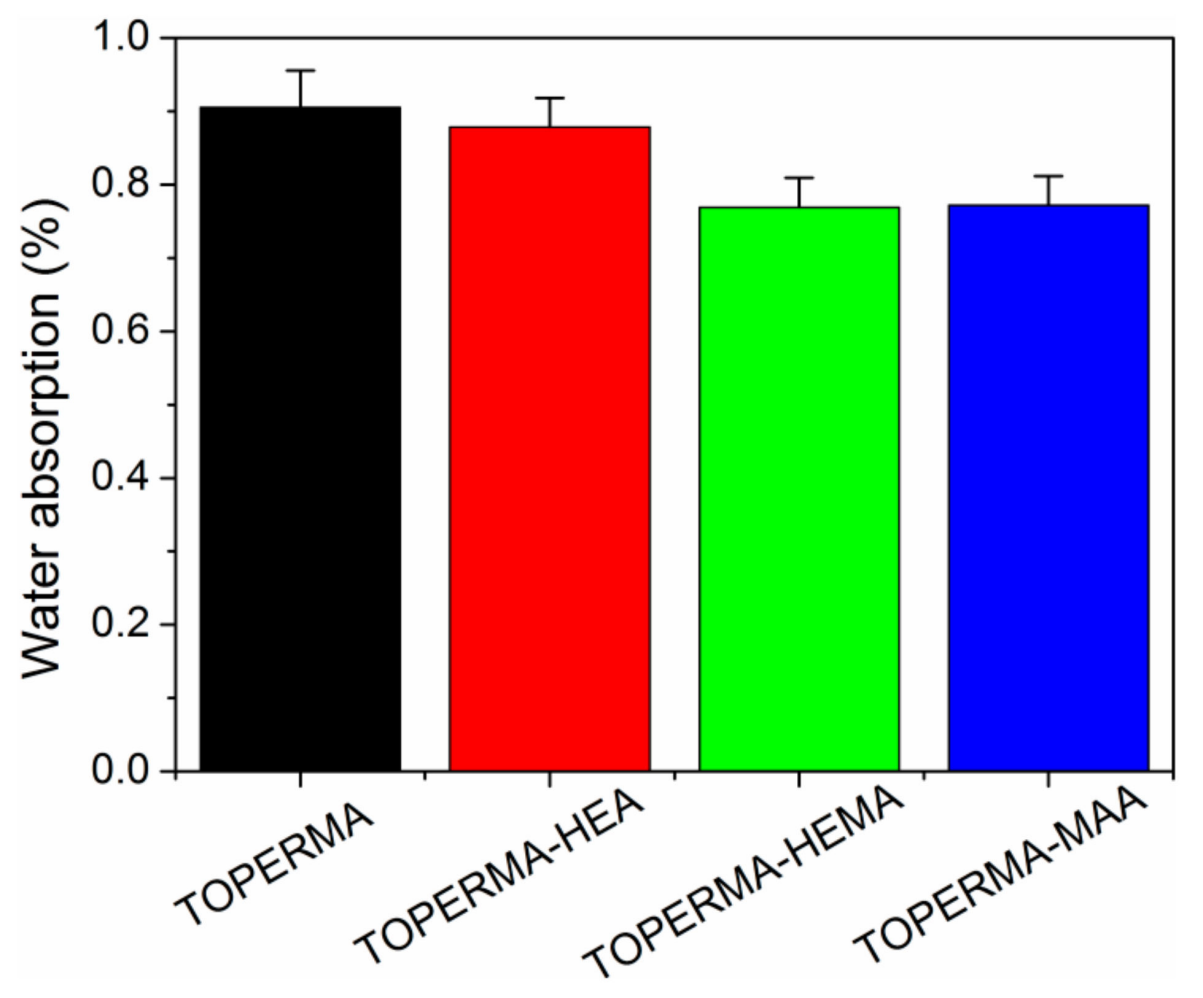
Water absorption of the cured TO-based resins.

The values of water absorption for the cured TO-based maleate and Co-UE resins are illustrated in Figure 6. Firstly, all of the materials showed a very low water absorption (<1%), which is probably because TO is a drying oil. Second, all of the TO-based Co-UE resins demonstrated lower water uptakes than the TOPERMA resin, which is possibly attributed to the higher *ν_e_* values of the resulting Co-UE thermosets and the hydrophobic structures from the unsaturated alcohols. In addition, compared with the TOPERMA-HEA resin, the TOPERMA-HEMA and TOPERMA-MAA resins also possessed lower water absorption, indicating that the employment of HEMA and MAA could further increase the hydrophobicity for the Co-UE materials.

## 4. Conclusions

For plant oil-based UE thermosets, improving their ultimate properties, such as stiffness and *T*_g_, is closely related to their real application in the area of structural plastics. Previous studies have suggested that the development of UE macromonomers or oligomers with high C=C functionality could lead to biobased materials with high crosslink density and good ultimate properties. This work also showed that the developed TO-based, Co-UE resins obviously possessed higher C=C functionality and better performance in stiffness and *T*_g_ than the original TO-based maleate resin. However, the previous literature did not show us how to further improve their performance for these new oil-based UE resins. In this work, we successfully found out some new ways to improve their performance. The first way is to incorporate the structure of steric hindrance into the TO-based Co-UE macromonomers. For example, the TOPERMA-HEMA resin showed a tensile strength of 30.9 MPa and Young’s modulus of 2.12 GPa, which was 2.0% and 5.4% higher than those of TOPERMA-HEA resin. The second way is to reduce the length of the flexible chain in the Co-UE macromonomers. For instance, the TOPERMA-MAA resin showed a tensile strength of 32.2 MPa, Young’s modulus of 2.38 GPa, and *T*_g_ of 130.3 °C, which was 4.2%, 12.3%, and 4.2% larger than those of TOPERMA-HEMA resin, respectively. Moreover, a combined effect could be achieved when the two ways were applied simultaneously into modulating the structure of the Co-UE monomer, e.g., the tensile strength and Young’s modulus of the TOPERMA-MAA resin increased by 6.3% and 18.4% compared with the TOPERMA-HEA resin. In general, this fundamental research could provide effective guidance for the preparation of plant oil-based UE thermosets with high performance.

## Figures and Tables

**Table 1:** Data of Characterization of TOPERMA and TO-based Co-UE Monomers.

Sample ID	*M_w_*^a^ (g/mol)	*M_n_*^b^ (g/mol)	*D* ^c^	*N*_C=C_^d^
TOPERMA	2247	1043	2.15	1.26
TOPERMA-HEA	3396	1406	2.42	1.62
TOPERMA-HEMA	3099	1298	2.39	1.56
TOPERMA-MAA	3120	1313	2.38	1.61

^a^ Weight-average molar mass. ^b^ Number-average molar mass. ^c^ Polydispersity index. ^d^ Introduced C=C functionality per fatty acid.

**Table 2:** Dynamic mechanic and thermal properties of the cured TO-based resins.

Samples	*E*′_25_*^a^* (GPa)	*T*_g_*^b^* (°C)	*ν*_e_*^c^* (10^3^ mol/m^3^)	*T*_5_*^d^* (°C)	*T*_p1_*^e^* (°C)	*T*_p2_*^e^* (°C)	*w_char_^f^* (%)
TOPERMA	2.05	123.1	3.44	384.9	408.0	453.8	9.04
TOPERMA-HEA	2.07	125.8	4.28	380.2	413.0	455.1	8.45
TOPERMA-HEMA	2.11	125.0	3.75	370.8	403.8	442.7	10.2
TOPERMA-MAA	2.33	130.3	3.93	379.5	406.4	452.3	8.36

*^a^* Storage modulus at 25 °C. *^b^* Glass transition temperature. *^c^* Crosslink density. *^d^* 5% weight loss temperature. *^e^* Peak temperature at the curves of weight loss rate; two peak values were observed for all the samples. *^f^* Char yield.

**Table 3:** Mechanical properties of the cured TO-based resins.

Samples	*σ ^a^* (MPa)	*E ^b^* (GPa)	*ε ^c^* (%)
TOPERMA	27.4 ± 2.2	1.91 ± 0.02	4.03 ± 0.29
TOPERMA-HEA	30.3 ± 2.2	2.01 ± 0.06	3.98 ± 0.11
TOPERMA-HEMA	30.9 ± 1.7	2.12 ± 0.03	3.11 ± 0.04
TOPERMA-MAA	32.2 ± 1.0	2.38 ± 0.15	3.79 ± 0.25

*^a^* Tensile strength. *^b^* Young’s modulus. *^c^* Tensile breaking strain.

## Supplementary Materials

The Supporting Information is available free of charge on the MDPI Publications website at https://www.mdpi.com/2073-4360/11/5/826/s1.

## References

[1] Kandelbauer A., Tondi G., Zaske O.C., Goodman S.H., Dodiuk H., Goodman S.H. (2014). Unsaturated polyesters and vinyl esters. Handbook of Thermoset Plastics.

[2] Barile C., Casavola C., De Cillis F. (2019). Mechanical comparison of new composite materials for aerospace applications. Compos. Part B-Eng..

[3] Liu C.G., Wang C.N., Tang J.J., Zhang J., Shang Q.Q., Hu Y., Wang H.X., Wu Q., Zhou Y.H., Lei W. (2018). High-performance biobased unsaturated polyester nanocomposites with very low loadings of graphene. Polymers.

[4] Kandola B.K., Ebdon J.R., Chowdhury K.P. (2015). Flame retardance and physical properties of novel cured blends of unsaturated polyester and furan resins. Polymers.

[5] Raquez J.M., Deleglise M., Lacrampe M.F., Krawczak P. (2010). Thermosetting (bio)materials derived from renewable resources: A critical review. Prog. Polym. Sci..

[6] Chen J.Q., Tang C.Q., Yue Y.Y., Qiao W.C., Hong J.G., Kitaoka T., Yang Z. (2017). Highly translucent all wood plastics via heterogeneous esterification in ionic liquid/dimethyl sulfoxide. Ind. Crop. Prod..

[7] Guo Y., Chen J.Q., Su M., Hong J.G. (2018). Bio-based plastics with highly efficient esterification of lignocellulosic biomass in 1-methylimidazole under mild conditions. J. Wood Chem. Technol..

[8] Feng Y.C., Liang H.Y., Yang Z.M., Yuan T., Luo Y., Li P.W., Yang Z.H., Zhang C.Q. (2017). A solvent-free and scalable method to prepare soybean-oil-based polyols by thiol-ene photo-click reaction and biobased polyurethanes therefrom. ACS Sustain. Chem. Eng..

[9] Zhang C.Q., Garrison T.F., Madbouly S.A., Kessler M.R. (2017). Recent advances in vegetable oil-based polymers and their composites. Prog. Polym. Sci..

[10] Chen J.Q., Su M., Zhang X.L., Chen R.P., Hong J.G., Yang L.Y., Yang Z. (2014). The role of cations in homogeneous succinoylation of mulberry wood cellulose in salt-containing solvents under mild conditions. Cellulose.

[11] Huang C., Dong H., Su Y., Wu Y., Narron R., Yong Q. (2019). Synthesis of carbon quantum dot nanoparticles derived from byproducts in bio-refinery process for cell imaging and in vivo bioimaging. Nanomaterials.

[12] Huang C., Su Y., Shi J., Yuan C., Zhai S., Yong Q. (2019). Revealing the effects of centuries of ageing on the chemical structural features of lignin in archaeological fir woods. New J. Chem..

[13] Wool R.P., Sun X.S. (2005). Bio-Based Polymers and Composites.

[14] Liu W., Fei M.-E., Ban Y., Jia A., Qiu R. (2017). Preparation and evaluation of green composites from microcrystalline cellulose and a soybean-oil derivative. Polymers.

[15] Costa C., Fonseca A.C., Moniz J., Godinho M., Serra A.C., Coelho J.F.J. (2016). Soybean and coconut oil based unsaturated polyester resins: Thermomechanical characterization. Ind. Crop Prod..

[16] Qin Y., Jia J., Zhao L., Huang Z., Zhao S., Zhang G., Dai B., Chen R., Sung W.P. (2012). Synthesis and characterization of soybean oil based unsaturated polyester resin. Biotechnology, Chemical and Materials Engineering, Pts 1-3.

[17] Khot S.N., Lascala J.J., Can E., Morye S.S., Williams G.I., Palmese G.R., Kusefoglu S.H., Wool R.P. (2001). Development and application of triglyceride-based polymers and composites. J. Appl. Polym. Sci..

[18] Can E., Kusefoglu S., Wool R.P. (2001). Rigid, thermosetting liquid molding resins from renewable resources. I. Synthesis and polymerization of soy oil monoglyceride maleates. J. Appl. Polym. Sci..

[19] Can E., Wool R.P., Kusefoglu S. (2006). Soybean and castor oil based monomers: Synthesis and copolymerization with styrene. J. Appl. Polym. Sci..

[20] Eren T., Kusefoglu S.H. (2005). Synthesis and polymerization of the acrylamide derivatives of fatty compounds. J. Appl. Polym. Sci..

[21] Eren T., Kusefoglu S.H. (2004). Synthesis and polymerization of the bromoacrylated plant oil triglycerides to rigid, flame-retardant polymers. J. Appl. Polym. Sci..

[22] Lu J., Khot S., Wool R.P. (2005). New sheet molding compound resins from soybean oil. I. Synthesis and characterization. Polymer.

[23] Echeverri D.A., Rios L.A., Rivas B.L. (2015). Synthesis and copolymerization of thermosetting resins obtained from vegetable oils and biodiesel-derived crude glycerol. Eur. Polym. J..

[24] Zhang P., Zhang J.W. (2013). One-step acrylation of soybean oil (so) for the preparation of so-based macromonomers. Green Chem..

[25] Liu C.G., Dai Y., Hu Y., Shang Q.Q., Feng G.D., Zhou J., Zhou Y.H. (2016). Highly functional unsaturated ester macromonomer derived from soybean oil: Synthesis and copolymerization with styrene. ACS Sustain. Chem. Eng..

[26] Gomez C.L., Echeverri D.A., Inciarte H.C., Rios L.A. (2019). Efficient processing of bioglycerol to a novel biobased polyunsaturated monomer. J. Chem. Technol. Biotechnol..

[27] Liu C.G., Shang Q.Q., Jia P.Y., Dai Y., Zhou Y.H., Liu Z.S. (2016). Tung oil-based unsaturated co-ester macromonomer for thermosetting polymers: Synergetic synthesis and copolymerization with styrene. ACS Sustain. Chem. Eng..

[28] Liu C.G., Yang X.H., Cui J.F., Zhou Y.H., Hu L.H., Zhang M., Liu H.J. (2012). Tung oil based monomer for thermosetting polymers: Synthesis, characterization, and copolymerization with styrene. Bioresources.

[29] Liu C.G., Zhou Y.H., Cheng R.S. (2013). Quantitative characterization of complex formation of a pmma/peg solution by sec-ls. J. Liq. Chromatogr. Relat. Technol..

[30] Can E., Wool R.P., Küsefoğlu S. (2006). Soybean- and castor-oil-based thermosetting polymers: Mechanical properties. J. Appl. Polym. Sci..

[31] Andjelkovic D.D., Valverde M., Henna P., Li F., Larock R.C. (2005). Novel thermosets prepared by cationic copolymerization of various vegetable oils—Synthesis and their structure–property relationships. Polymer.

