# Supplementary material for: Synthesis and Properties of Tung Oil-Based Unsaturated Co-Ester Resins Bearing Steric Hindrance
Source: Polymers (Basel). 2019 May 7;11(5):826. doi: 10.3390/polym11050826 (PMC6572467; doi:10.3390/polym11050826)
Supplement: Supplementary file 1 [file polymers-11-00826-s001.pdf]

Supporting Information (SI)

Synthesis and properties of tung oil-based unsaturated co-ester resins bearing steric hindrance

Chengguo Liu[a, *], Qiong Wu[a,b], Rongrong An[c], Qianqian Shang[a], Guodong Feng[a], Yun Hu[a], Puyou Jia[a], Yonghong Zhou[a, *], Wen Lei[b,*]

[a] Institute of Chemical Industry of Forest Products, Chinese Academy of Forestry; National Engineering Lab for Biomass Chemical Utilization; Key Lab on Forest Chemical Engineering, State Forestry Administration; Key Lab of Biomass Energy and Material, Jiangsu Province; Nanjing 210042, P. R. China

[b] College of Science, Nanjing Forestry University, Nanjing 210037, P. R. China

[c] College of Geographic and Biologic Information, Nanjing University of Posts and Telecommunications, Nanjing 210023, P. R. China

***** Correspondence: **Corresponding authors**: Chengguo Liu. E-mail: liuchengguo@icifp.cn; Tel.: +86-25-85482520; Fax: +86-25-85482520; Yonghong Zhou. E-mail: zyh@icifp.cn; Tel.: +86-25-854825777; Fax: +86-25-854825777; Wen Lei. E-mail: njfuleiwen@163.com; Tel.: +86-25-85427206; Fax: +86-25-85427206.

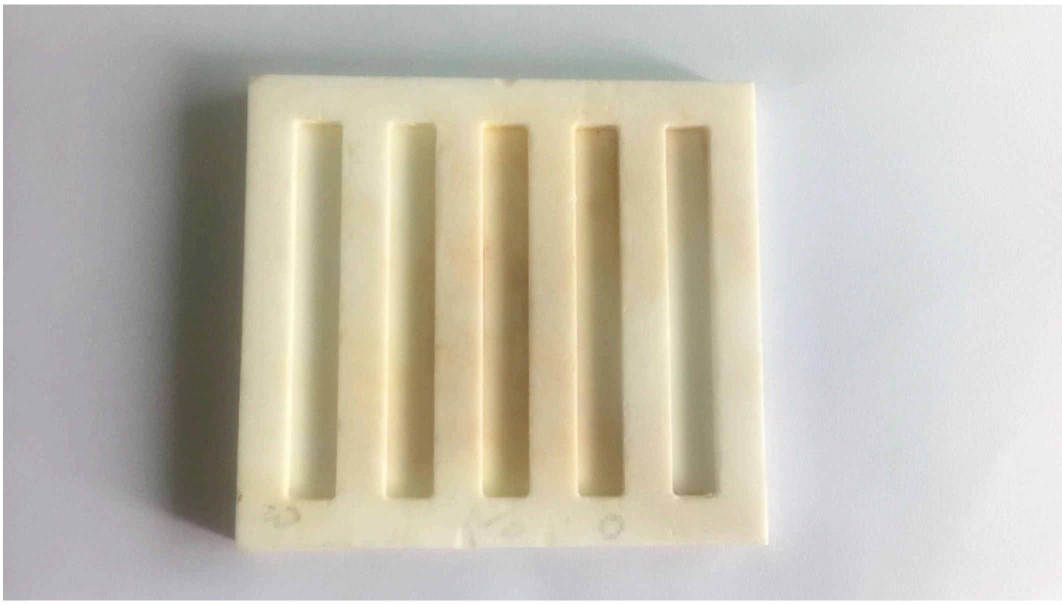

Fig. S1 PTFE mold used for preparing samples of DMA test (Shape: Cuboid; Size: 80×10×4 mm^3)

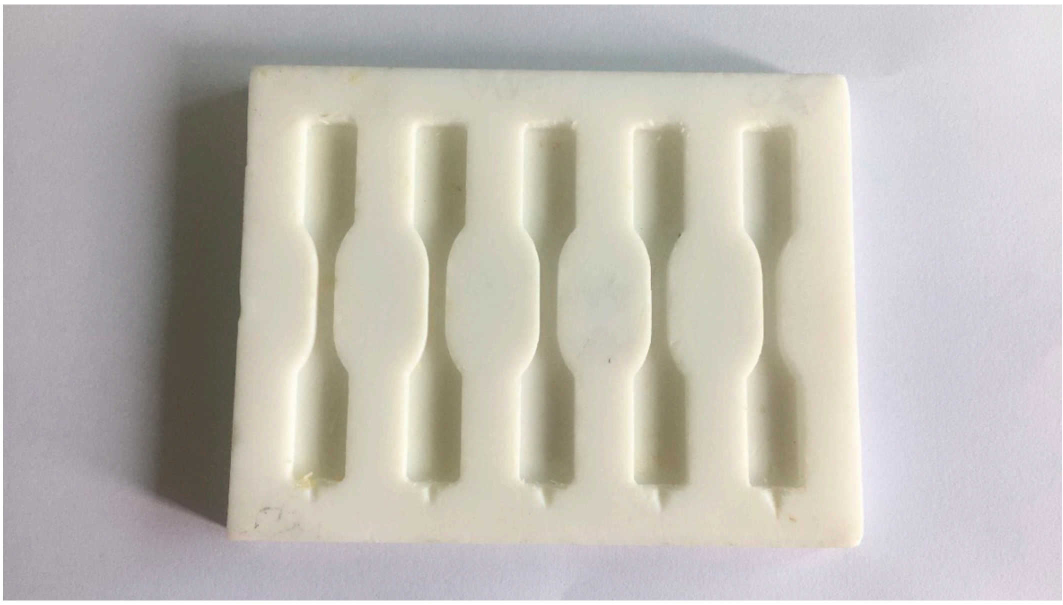

Fig. S2 PTFE mold used for preparing samples of tensile property test (Shape: Dumbbell; Length overall: 63.5 mm; Width overall: 9.53 mm; Size of the narrow middle part: 9.53×3.18×3.2 mm^3)
